# Periodontitis and Dementia: A Bidirectional Relationship?

**DOI:** 10.1177/00220345211043461

**Published:** 2021-10-16

**Authors:** A. Harding, S.K. Singhrao

**Affiliations:** 1 Dementia and Neurodegenerative Diseases Research Group, Faculty of Clinical and Biomedical Sciences, School of Dentistry, University of Central Lancashire, Preston, UK

**Keywords:** Alzheimer’s disease, risk factor, causal relationship, periodontal disease, oral health, apolipoprotein E allele 4 susceptibility gene

A body of scientific evidence supports the view that periodontal disease and Alzheimer’s disease (AD) are comorbid. While periodontal disease affects tooth-supporting tissues and the host’s immune responses, leading to eventual tooth loss, AD is characterized by 2 histologic diagnostic markers at autopsy: the extraneuronal amyloid plaques and the intraneuronal neurofibrillary tangles (Hyman et al. 2012). Other lesions without a role in the neuropathologic diagnosis of AD include neuronal and synaptic loss, neuroinflammation, and cerebral amyloid angiopathy (Dugger and Dickson 2017), which are of importance in understanding the disease process. Understanding the relationship between AD and periodontitis is hindered by the long-standing dogma that those with dementia or AD-associated dementia are at greater incidence of longitudinally manifesting periodontal disease than those without it. This view clearly assumes that AD-associated dementia is a risk factor for periodontal disease. The assumption would be that the behavioral changes associated with the onset of dementia, namely poorer levels of oral hygiene, are the predominant cause rendering an individual with AD more susceptible to periodontal disease. This currently held dogmatic view is behind the proposal of a bidirectional relationship between AD and periodontitis! Given that a growing body of literature (Stein et al. 2007; Sparks Stein et al. 2012; Farhad et al. 2014; Demmer et al. 2020; Nadim et al. 2020) suggests that periodontal disease can precede AD-associated dementia manifestation, the suggestion is that a converse relationship also exists. This was something that Ma et al. (2021) also identified in their cases over the course of their study, but they correctly rejected these cases due to the possibility of confounding factor interferences. To this end, Ma et al. assessed the effect of AD without diverse pathologies that may act as confounding factors on conditions that develop because of the human aging process.

Ma et al. (2021) were able to clarify the plausibility of a bidirectional relationship in their population-based cohort study. It is interesting that the incidence of the bidirectional relationship that Ma et al. identified appears to be associated with the younger patients with AD (<60 y) and in the 61- to 70-y cohort with AD, who are likely to have a longer duration of the mental illness. The bidirectional relationship also exists in the higher age group (>80 y) with AD-associated dementia, but this group shows less severe periodontal disease. A reason behind this could have been the result of lifestyle factors—for example, insomnia, stress, or other confounding factors that were not identified or considered in this study. Although Ma et al. did not report whether their younger cohorts had known genetic susceptibilities or whether there were mutated genes in the selected AD cases, it is plausible to suggest that genetics played a role in the early-onset AD or inherited form of AD and not in AD cases with as-yet-unidentified genetic vulnerabilities that are categorized into the sporadic or late-onset AD form.

This brings us to the apolipoprotein E allele 4 susceptibility gene inheritance (Corder et al. 1993), as it 1) represents the second-highest risk factor for AD-associated dementia after advancing age, 2) has the implication for an early clinical manifestation of the disease, and 3) has potential relevance for the interaction between this susceptibility gene and infection. In addition, there are known genetic vulnerabilities associated with the inherited or early-onset form of AD, and these include the amyloid precursor protein and presenilin 2. These act to enhance amyloid precursor protein gene processing to promote enhanced amyloid deposition (Selkoe 2001), which again may have played a role for the severity of periodontal disease in the younger patients, as often seen in the dental clinic. It is well documented that, although less common, there are groups of younger patients who have far more aggressive periodontal disease, and this has genetic, racial, and immunologic associations (Merchant et al. 2014).

An alternative view would be that a subset of periodontal patients with subsequent AD development denotes a subset of AD cases where the relationship is likely to be the reverse. In a letter to the *British Dental Journal*, Harding and Singhrao (2019) argued that the evidence is lacking to support a generalization in terms of a bidirectional relationship in all AD cases. It is encouraging that Ma et al. (2021) finally made significant progress in their study to find the actual subset of AD-associated dementia cases with longitudinal periodontal disease, in which they identified a plausible bidirectional relationship. A similar study will benefit from revealing whether periodontal disease leads to AD-associated dementia development and results in an equivalent subset of AD cases, as postulated by Harding and Singhrao.

Long-standing poor oral hygiene/pathogen load (as reflected by increased pocket depth) is a likely risk for exacerbating early-onset AD and rapid deterioration in cognition due to peripheral and intracerebral inflammation following periodontal disease. Ma et al. (2021) concluded that a convincing causal relationship of AD-associated dementia with periodontal disease exists. However, determining causality in studies on oral-systemic (Raittio and Farmer 2021) and oral-chronic cognitive diseases such as AD-associated dementia is complex due to the nature of human pathophysiology and an individual’s lifestyle, genes, and environmental influences—all of which make reaching this simplistic conclusion a challenge. Although current research does indicate a potential dependency/relationship between periodontal disease and AD-associated dementia, further research is required to clarify the directionality of this association.
